# miRNA‐27b levels are associated with CYP3A activity in vitro and in vivo

**DOI:** 10.1002/prp2.192

**Published:** 2015-10-27

**Authors:** Lena Ekström, Ilona Skilving, Marie‐Louise Ovesjö, Eleni Aklillu, Hanna Nylén, Anders Rane, Ulf Diczfalusy, Linda Björkhem‐Bergman

**Affiliations:** ^1^ Division of Clinical PharmacologyDepartment of Laboratory MedicineKarolinska InstitutetKarolinska University Hospital, HuddingeSE‐141 86StockholmSweden; ^2^ Division of Clinical ChemistryDepartment of Laboratory MedicineKarolinska InstitutetKarolinska University Hospital, HuddingeSE‐141 86StockholmSweden; ^3^ Division of Clinical MicrobiologyDepartment of Laboratory MedicineKarolinska InstitutetKarolinska University Hospital, HuddingeSE‐141 86StockholmSweden; ^4^ Palliative Home Care and Hospice WardASIH Stockholm SödraLångbro ParkBergtallsvägen 12SE‐125 59ÄlvsjöSweden

**Keywords:** 4*β*–hydroxycholesterol, 6*β*‐hydroxylation of testosterone, CYP3A‐activity, cytochrome P450 3A4, drug metabolism, microRNA, miR‐27b, N‐demethylation of dextromethorphan, PPAR*α*, VDR, vitamin D

## Abstract

Previous in vitro studies have shown that microRNA‐27b (miR‐27b) may regulate mRNA levels of CYP3A4, vitamin D receptor (VDR), and Peroxisome proliferator‐activated receptor *α* (PPAR *α*) as well as CYP3A4 protein expression and activity. In vitro studies have also shown that vitamin D may affect the expression of CYP3A4. The primary aim of this pilot study was to investigate the association between miR‐27b and CYP3A expression and activity. The secondary aim was to investigate the association between 25‐hydroxy vitamin D in serum and CYP3A activity. Mi‐RNA‐27b was quantified using real‐time PCR in serum samples (*n* = 28) and 25‐hydroxyvitamin D was measured and correlated with the levels of the endogenous CYP3A activity marker 4*β*‐hydroxycholesterol. In addition, the correlation between miR‐27b and CYP3A activity, measured by dextromethorphan N‐demethylation and 6*β*‐hydroxylation of testosterone and the gene expression of CYP3A4, VDR and PPAR *α* were assessed in 20 human liver samples. A significant association between circulatory miR‐27b levels and 4*β*‐hydroxycholesterol ratio was found; *P* = 0.04, and between hepatic miR‐27b levels and CYP3A activity, measured by dextromethorphan N‐demethylation in human liver (*P* = 0.04). There was no association between hepatic miR‐27b and mRNA levels of CYP3A4, VDR or PPAR *α*. There was a significant association between serum 25‐hydroxyvitamin D levels and 4*β*‐hydroxycholesterol ratio, *P* = 0.002. In conclusion, this pilot‐study supports the hypothesis that miR‐27b levels as well as 25‐hydroxyvitamin D may affect CYP3A activity in vivo. The results indicate that miR‐27b exerts its inhibitory effect on a translational level rather than affecting mRNA levels.

**Abbreviations**
4*β*‐hydroxycholesterol ratio: 4*β*–hydroxycholesterol/cholesterol ratio × 10^4^
CI: confidence interval
CYP: cytochrome P450
miR‐27b: microRNA‐27b
PPAR *α*: peroxisome proliferator‐ activated receptor *α*
VDR: vitamin D receptor

## Introduction

Cytochrome P450 3A (CYP3A) enzymes constitute the most important drug metabolizing enzymes, but are also involved in the metabolism of endogenous compounds, such as steroid substrates (Daly 2006; Luoma 2008). The enzyme activity is highly variable between individuals and the activity may also vary within the same individual at different time points. The intra‐individual variation can be due to drugs or food that affects the expression and/or activity of the enzyme. One such possible regulation is vitamin D that has been shown to affect the expression of CYP3A4 in vitro (Schmiedlin‐Ren et al. 1997) although the results from in vivo studies are conflicting (Lindh et al. 2011; Thirumaran et al. 2012; Björkhem‐Bergman et al. 2013b; Nylén et al. 2014). One explanation for these discrepancies might be that intestinal CYP3A activity is more affected by vitamin D (Thirumaran et al. 2012) than the hepatic enzymes (Björkhem‐Bergman et al. 2013b; Nylén et al. 2014).

The inter‐individual variation in CYP3A activity has been shown in part to be determined by genetic variations such as SNPs in the *CYP3A4* gene (Antunes et al. 2015; Pallet et al. 2015) as well as in regulatory nuclear factors (Lunde et al. 2014). In addition to genetic polymorphisms, epigenetics factors, which refer to DNA methylation, histone acetylation and regulation by microRNA (miRNA) may alter CYP metabolism (Ingelman‐Sundberg et al. 2007). miRNA is a family of short, noncoding RNA molecules that are highly abundant in the circulation. Several miRNAs have been proposed as disease biomarkers for diagnosis, prognosis and treatment outcomes, particularly in cancer (reviewed by Witwer (2015)). miRNAs pair with complementary mRNAs to direct mRNA degradation and/or translational repression. It has been shown that miRNA‐27b (miR‐27b) target sequences in *CYP3A4*, as well as the genes of the nuclear factors vitamin D receptor (*VDR*)*,* retinoid X receptor *α* (*RXRα*), and peroxisome proliferator‐activated receptor *α* (*PPAR α*) and negatively regulate CYP3A4 mRNA and protein expression in vitro (Pan et al. 2009; Kida et al. 2011). Li et al. (2014) showed that the presence of recombinant miR‐27b led to lower midazolam 1’‐hydroxylase activity in an intestinal human colon adenocarcinoma cell line. However, to our knowledge it has never been investigated if miR‐27b is associated with CYP3A4 activity in vivo.

4*β*‐hydroxycholesterol is formed from cholesterol by CYP3A4 and CYP3A5 and can be used as a marker of CYP3A activity (Diczfalusy et al. 2009). Several studies have shown that 4*β*‐hydroxycholesterol is as good as midazolam hydroxylation in monitoring CYP3A‐induction (Björkhem‐Bergman et al. 2013a; Shin et al. 2013; Kasichayanula et al. 2014). Although the marker might be less suitable for monitoring inhibition of enzyme activity due to its long half‐life, we have shown that it is possible to detect strong inhibition of CYP3A if the inhibition last for at least 1 week (Lütjohann et al. 2009). Moreover, in a recent study in patients with impaired liver function, it is shown that there is a high accordance between 4*β*‐hydroxycholesterol and midazolam hydroxylation in predicting CYP3A activity also when the CYP3A activity is reduced (Woolsey et al. 2015).

In liver tissue, determination of dextromethorphan N‐demethylation and 6*β*‐hydroxylation of testosterone are well‐known markers for measuring CYP3A activity (Waxman et al. 1983; Al‐Jenoobi et al. 2015).

The primary aim of this pilot study was to investigate the association between miR‐27b and CYP3A expression and activity and miR‐27b and genes regulating mRNA levels of CYP3A4. The secondary aim was to investigate the association between 25‐hydroxyvitamin D in serum and CYP3A activity.

## Materials and Methods

### Study populations

We analyzed serum samples from a previously performed clinical study in 180 statin‐treated patients in the primary health care of Stockholm. Written informed consent was obtained from all participants. The study was approved by the local Ethics Committee (Dnr: 2006/431‐31/2) and was performed in accordance with the declaration of Helsinki. The study is described in detail in another article (Skilving et al. 2015). From the original study, 28 patients were analyzed for 4*β*‐hydroxycholesterol levels in their baseline blood samples taken at study inclusion.

Human adult liver specimens were collected from the liver bank at the Department of Clinical Pharmacology, Karolinska University Hospital Huddinge, which was first established in 1980 as described previously (von Bahr et al. 1980; Ghotbi et al. 2009). The samples used in this study were collected between 1997 and 2001 and comprised 20 liver samples from patients 18–65 years of age, males (*n* = 7), females (*n* = 10), or unknown sex (*n* = 3).

### Determination of 4β‐hydroxycholesterol

The oxysterol 4*β*‐hydroxycholesterol was measured as described previously (Bodin et al. 2001; Diczfalusy et al. 2011). Briefly, isotope dilution gas chromatography‐mass spectrometry was used and deuterium labeled 4*β*‐hydroxycholesterol was used as internal standard. The within‐day variation was 4.5% and the between‐day variation was 8.2% at 25 ng/mL. The method was linear upto 600 ng/mL. There is a weak but significant correlation between plasma 4*β*‐hydroxycholesterol and plasma total cholesterol (Diczfalusy et al. 2008). In longitudinal studies, we have therefore used the molar ratio of 4*β*‐hydroxycholesterol/cholesterol × 10^4^ (4*β*‐hydroxycholesterol ratio) to account for differences in plasma cholesterol with time. Cholesterol was determined by a commercial enzymatic method (Cholesterol CHOD‐PAPP, Roche Diagnostics GmbH, Mannheim, Germany) run on a Roche/Hitachi Modular instrument. The Cv was 1.3% (at 5 mmol/L).

### miR‐27b analysis

Serum samples from 28 patients were used to extract miRNA, using miRNeasy Kit (Qiagen, Hilden, Germany), whereas total RNA from liver samples has previously been isolated, using RNeasykit (Qiagen). TaqMan probe‐based qRT‐PCR (Life Technology, Foster City CA USA) was performed according to the manufacturer's protocol. Briefly, 5–20 ng of RNA was reversed transcribed to complementary DNA (cDNA) using MicroRNA Reverse Transcription Kit (Life Technologies) and a stem‐loop primer of U6 snRNA and hsamiR‐27b (Life Technologies ID #001973, and 000409, respectively). Real‐time PCR was carried out using corresponding U6snRNA and hsamir‐R‐27b gene‐specific assays and TaqMan^®^ Universal Master Mix II no UNG (Life Technologies) on the Fast 7500 (Applied Biosystems, Foster City, CA). Delta Ct was calculated according to (Schmittgen and Livak 2008) using U6‐snRNA as a control‐gene and randomly choosing a sample as a calibrator in each study population.

### 25‐hydroxyvitamin D levels

Levels of 25‐hydroxyvitamin D in serum were determined by a commercial immunochemical method, LIAISON 25 OH Vitamin D TOTAL Assay (DiaSorin S.p.A., Saluggia, Italy), detectable range 7.5–175 nmol/L, CV 2–5%, at the Department of Clinical Chemistry, Karolinska University Hospital.

### RNA extraction and cDNA synthesis in liver tissue

Total RNA from 200 mg of human liver tissue was prepared using RNeasykit (Qiagen) according to the manufacturer's protocols. Reverse transcription was performed on 0.3–0.5 *μ*g RNA samples. Master mix with RNase inhibitor was prepared according to High‐Capacity cDNA Reverse Transcription Kits Protocol (Applied Biosystems) and was performed using PCR System 2700(Applied Biosystems) under conditions 25°C for 10 min, 37°C for 120 min, 85°C for 5 min, and 4°C overnight.

### Q‐PCR gene expression

CYP3A4, VDR, and PPAR*α* mRNA expression in the human liver samples had been determined in a previous study (Betts et al. 2015). In brief, PCR Master mix was prepared, Taq Man 2XPCR mix, 20X gene assay, 1 *μ*L cDNA in a final volume of 15 *μ*L. TaqMan^®^ Gene Expression Assays (Applied Biosystems) used were CYP3A4 (Hs00604506_m1), VDR (Hs00172113_m1), PPAR*α* (Hs00947536_m1) and 18S (4310893E‐1105050). Relative expression was calculated according to the 2‐ΔΔCt formula (Livak and Schmittgen 2001), using the housekeeping gene 18S as the internal control.

### Determination of dextromethorphan N‐demethylation and 6*β*‐hydroxylation of testosteron

The CYP3A activity in the liver tissues from the liver donor bank had been determined at the time they were collected, that is, 1997–2001. The methods used at this time point were measurements of demethylation of dextromethorphan and 6*β*‐hydroxylation of testosterone. The conversions of dextromethorphan by CYP3A catalyzed N‐demethylation to 3‐methoxymorphinan was measured as described elsewhere (Al‐Jenoobi et al. 2015). In brief, dextromethorphan solved in methanol at a final concentration of 25 *μ*mol/L was used and the incubation time was 30 minutes. The metabolite 3‐methoxymorphinan was detected with the HPLC method. The enzyme activity was expressed as pmol/min per mg protein.

Testosterone hydroxylation was determined as described by Waxman et al. (1983). Testosterone 50 nmol, dissolved in ethanol was used and the incubation time was 20 min. Enzyme activity was calculated and converted into pmoles of product based on the testosterone concentration in the incubation mixture and finally expressed as pmol/min per mg protein.

### Statistical analysis

All statistical tests were performed, using GraphPad Prism (San Diego, California, USA) v. 6.00 and values of *P* < 0.05 were considered statistically significant. For comparing men and women, unpaired two‐tailed *t*‐test was used since the data showed Gaussian distribution. To study the association between miR‐27‐b and 4*β*‐hydroxycholesterol, 25‐hydroxyvitamin D levels, 6*β*‐hydroxylation of testosterone, dextromethorphan N‐demethylation, gene expression in liver tissue and between 25‐hydroxyvitamin D levels and cholesterol, HDL, and Triglyceride levels, Spearman rank test was used since not all data were normally distributed.

## Results

### Circulatory miR‐27b, 25‐hydroxyvitamin D levels and CYP3A activity

**Figure 1:**
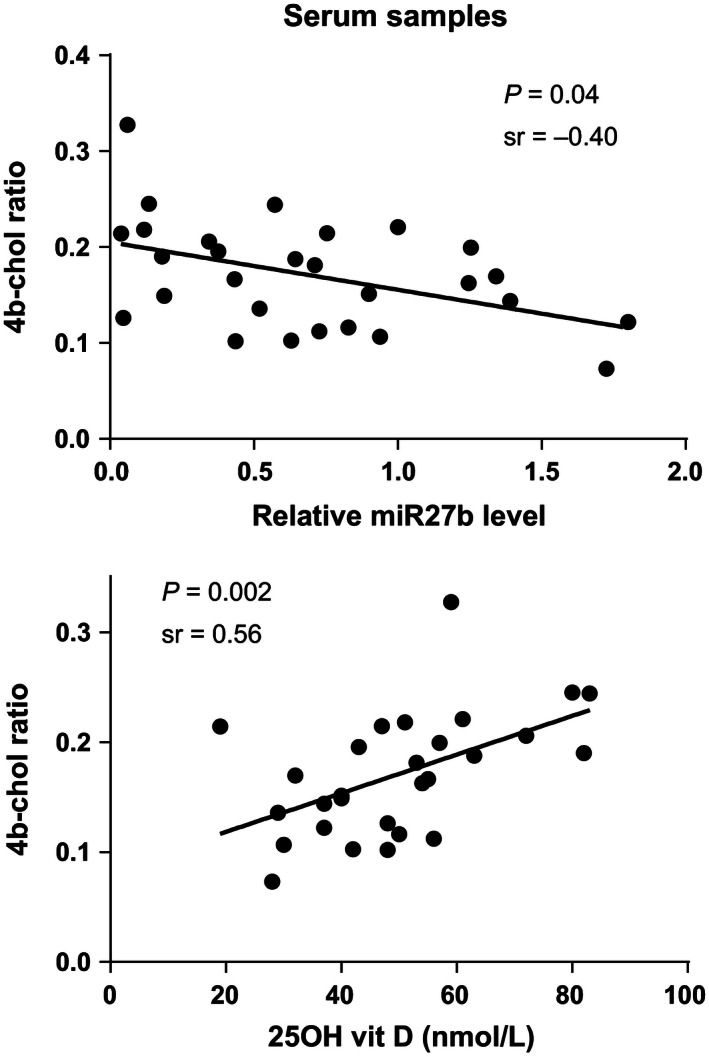
Association between relative miR‐27b levels and 4*β*‐hydroxycholesterol to cholesterol ratio (4b‐chol ratio) and 25‐hydroxyvitamin D levels (25‐OH vit D) and 4*β*‐hydroxycholesterol to cholesterol ratio in 28 patients, using Spearman's correlation. sr = Spearman's *r*.

To test the association between miR‐27b levels and CYP3A activity in vivo serum samples from a previously performed clinical study was used. Twenty‐eight patients were included in the study, 17 women and 11 men. The median age in the cohort was 64 years (range 27–84). There was a 50‐fold inter‐individual variation in circulatory level of miR‐27b in the patients (data not shown). There were no associations between miR‐27b and total cholesterol, TG and HDL concentrations (data not shown). Neither was there any association between miR‐27b levels and gender. A significant correlation between circulatory miR‐27b levels and 4*β*‐hydroxycholesterol ratio was found; Spearman's *r* = −0.40, *P* = 0.04 (Fig. 1).

To further study factors that may affect CYP3A‐activity, we studied the relationship between 25‐hydroxyvitamin D levels in blood and 4*β*‐hydroxycholesterol ratio. There was a significant association between 25‐hydroxyvitamin D levels and 4*β*‐hydroxycholesterol ratio, Spearman's *r* = 0.56, *P* = 0.002, (Fig. 1).

There was no correlation between 25‐hydroxyvitamin D levels in serum and cholesterol, HDL or triglycerid levels. The 25‐hydroxyvitamin D levels were similar in men and women; mean 49 nmol/L and 50 nmol/L, respectively.

### Hepatic miR‐27b and CYP3A4 mRNA expression

**Figure 2:**
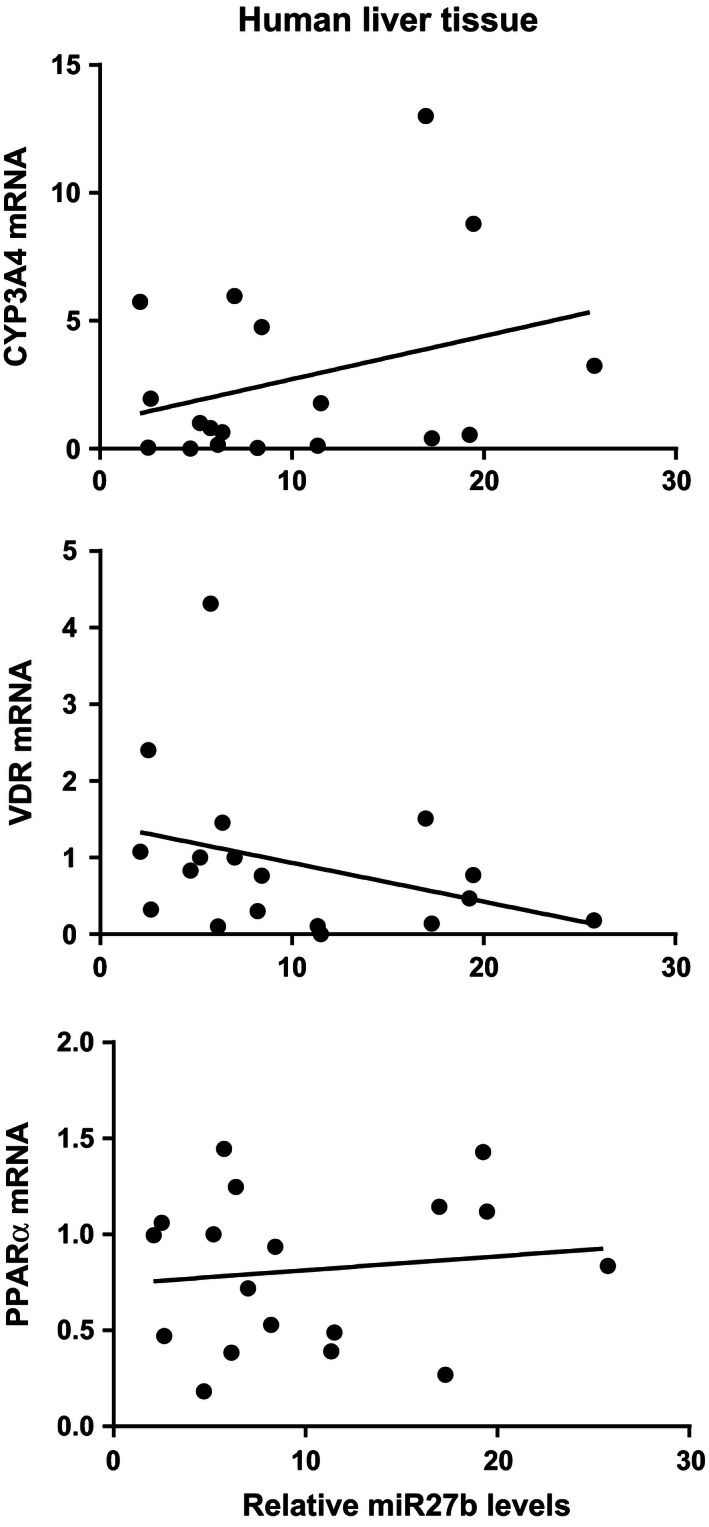
Association between relative hepatic levels (*n* = 20) of miR‐27b and mRNA‐levels of CYP3A4, vitamin D receptor (VDR) and peroxisome proliferator activated receptor *α* (PPAR *α*). There was no statistically significant correlation between the relative mRNA‐levels of CYP3A4 (Spearman's *r* = 0.26; *P* = 0.30), VDR (Spearman's *r* = −0.44; *P* = 0.07) or PPAR *α* (Spearman's *r* = 0.08; *P* = 0.75) and relative miR‐27b levels. Relative miR‐27b expression was calculated according to the 2‐*ΔΔ*Ct formula (Livak and Schmittgen 2001) using U6‐snRNA as an internal control.

There was a 50‐fold inter‐individual variation in the hepatic presence of miR‐27b, but no significant difference between women and men. There was no statistically significant correlation between mRNA‐levels of CYP3A4 (Spearman's *r* = 0.26; *P* = 0.30), VDR (Spearman's *r* = −0.44; *P* = 0.07), or PPAR*α* (Spearman's *r* = 0.08; *P* = 0.75) and miR‐27b levels as shown in Figure 2.

### Hepatic CYP3A‐activity and miR‐27b

**Figure 3:**
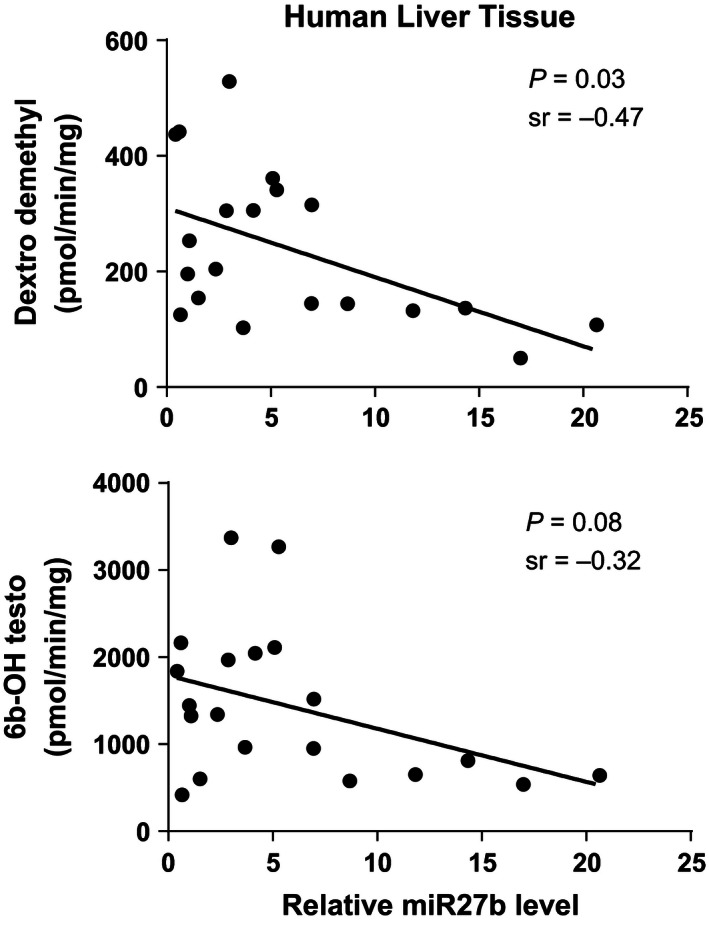
Association between relative hepatic levels (*n* = 20) of miR‐27b and CYP3A activity measured by N‐demethylation of dextromethorphan (dextro dimethyl) (pmol/min per mg); and 6*β*‐hydroxylation of testosterone (6b‐OH testo) (pmol/min per mg), using Spearman's correlation. sr = Spearman's *r*. Relative miR‐27b expression was calculated according to the 2‐*ΔΔ*Ct formula (Livak and Schmittgen 2001), using U6‐snRNA as an internal control.

CYP3A activity was measured in human liver tissue by demethylation of dextromethorphan and 6*β*‐hydroxylation of testosterone as shown in Figure 3. There was a significant correlation between miR‐27b levels and CYP3A activity measured by demethylation of dextromethorphan, Spearman's *r* = −0.47, *P* = 0.03. There was a tendency toward correlation between miR‐27b levels and CYP3A activity measured by 6*β*‐hydroxylation of testosterone, Spearman's *r* = −0.32, *P* = 0.08, but the results were not statistically significant.

## Discussion

In this study, we show for the first time that circulatory miR‐27b levels are associated with CYP3A activity, measured by the biomarker 4*β*‐hydroxycholesterol ratio, that is, lower miR‐27b levels are associated with higher CYP3A activity. This is in accordance with in vitro findings that have shown that miR‐27b down‐regulate CYP3A4 mRNA and protein expression in vitro (Pan et al. 2009). Furthermore, we also found a significant correlation between CYP3A activity in human liver tissue, measured by demethylation of dextromethorphan, and miR‐27b levels. However, we did not observe any correlation between miR‐27b and CYP3A4 gene expression in human liver samples. These results indicate that miR‐27b exerts its inhibitory effect on a translational level rather than affecting the mRNA levels. In addition to target CYP3A4, miR‐27b has been found to target nuclear factors such as VDR (Pan et al. 2009) and PPAR*α* (Kida et al. 2011). Here, we find no statistically significant correlations between miR‐27b and VDR and PPAR*α*, although a trend toward a negative correlation was found for VDR (*P* = 0.07), indicating that miR‐27b binding to VDR gene may degrade the mRNA transcript. Future studies with larger sample sizes will elucidate if the effect of miR‐27b on CYP3A4 activity is due to a direct inhibition of CYP3A4 expression or via regulation of nuclear factors.

There was a high inter‐individual variability in miR‐27b levels both in the liver and in the circulation. This is in agreement with Kida et al. (2011) who found a 70‐fold variation of miR‐27b in human liver samples. No difference in miR‐27b was seen between women and men. Notably, CYP3A4 expression and activity have in some studies been shown to be higher in women than in men (Wolbold et al. 2003; Björkhem‐Bergman et al. 2013b).

We have previously shown a seasonal variation in CYP3A‐dependent drug metabolism with higher CYP3A‐activity during the summer compared to the winter season (Lindh et al. 2011). We speculated that this variability could be due to seasonal variation in vitamin D levels since in vitro experiments have shown that vitamin D can induce CYP3A4 in vitro (Schmiedlin‐Ren et al. 1997). However, in two follow‐up studies, we could see no association between 25‐hydroxyvitamin D levels and the CYP3A‐marker 4*β*‐hydroxycholesterol (Björkhem‐Bergman et al. 2013b; Nylén et al. 2014). In this small study, a significant association was found. Due to the limited sample size, these results should be interpreted with caution.

Notably, there is additional support for miRNAs as important epigenetic factors, which may impact on drug metabolism. Thus, it has been speculated that miRNAs could be used as pharmacoepigenetic markers (Ingelman‐Sundberg et al. 2007). miR‐27b has been suggested to be a promising biomarker for different cancers (Lo et al. 2012; Ishihara et al. 2014) and cardiovascular disorders (Li et al. 2011). One drawback using miRNAs as biomarkers is that they often display large overlaps with various diseases and hence the specificity may be low (Witwer 2015). Further studies are warranted to study if miR‐27b determines the metabolic capacity of different CYP3A‐metabolized drugs and if it could be used as a marker for CYP3A activity.

This is to our knowledge the first study to investigate the impact of miR‐27b on CYP3A activity in vivo. However, an important limitation of this study is the small sample size. One of the CYP3A activity measurements, 6*β*‐hydroxylation of testosterone, only showed a tendency to correlate with miR‐27b levels that was not statistically significant, probably due to under‐powered sample size. Another limitation is the lack of data on the protein level of CYP3A. Instead a biomarker for CYP3A activity was used in serum and markers of CYP3A activity in liver tissue were used. Although 4*β*‐hydroxycholesterol has been shown to be as good as the measurement of midazolam hydroxylation in monitoring CYP3A‐induction, it might be less reliable in detecting reduced CYP3A levels due to the long half‐life of 4*β*‐hydroxycholesterol (Diczfalusy et al. 2009).

In conclusion, this pilot‐study supports the hypothesis that miR‐27b levels as well as 25‐hyrdoxyvitamin D may affect CYP3A activity in vivo and indicate that miR‐27b exert its inhibitory effect on a translational level rather than affecting the mRNA stability.

However, these results need to be confirmed in larger studies.

## Author Contributions

LE and LBB participated in research design;

LE, IS, MLO, EA, HN, AR, and UD conducted experiments;

LE, IS, MLO, HN, AR, and UD contributed new reagents or analytic tools;

LE and LBB performed data analysis; and

LE, IS, MLO, EA, HN, AR, UD, LBB wrote or contributed to the writing of the manuscript.

## Disclosures

None declared.
